# Supplementary material for: A machine learning prediction model for waiting time to kidney transplant
Source: PLoS One. 2021 May 20;16(5):e0252069. doi: 10.1371/journal.pone.0252069 (PMC8136711; doi:10.1371/journal.pone.0252069)
Supplement: S1 Table — (DOCX) [file pone.0252069.s001.docx]

**S1 Table. Characteristics of the cohort of patients on the kidney transplant waiting list at any time between January 1, 2000, and December 31, 2017**

|  | **Transplant**  **No (N=34421)** | | **Transplant**  **Yes (N=13732)** | **Total (N=48153)** | **p value** |
| --- | --- | --- | --- | --- | --- |
| **Age (years)** | |  |  |  | < 0.001^1^ |
| Mean (SD) | 50.40 (14.04) | | 44.13 (15.37) | 48.61 (14.71) |  |
| Median (Q1, Q3) | 52.00 (41.00, 61.00) | | 46.00 (34.00, 56.00) | 50.00 (39.00, 59.00) |  |
| Min - Max | 0.00 - 97.00 | | 0.00 - 82.00 | 0.00 - 97.00 |  |
| Missing | 0 | | 0 | 0 |  |
| **Time on Dialysis (months)** |  | |  |  | 0.002^1^ |
| Mean (SD) | 21.77 (30.34) | | 19.23 (26.05) | 21.05 (29.21) |  |
| Median (Q1, Q3) | 10.00 (5.00, 24.00) | | 10.00 (5.00, 22.00) | 10.00 (5.00, 23.00) |  |
| Min - Max | 0.00 - 180.00 | | 0.00 - 180.00 | 0.00 - 180.00 |  |
| Missing | 817 | | 519 | 1336 |  |
| **Race** |  | |  |  | < 0.001^2^ |
| Oriental | 697 (2.0%) | | 251 (1.8%) | 948 (2.0%) |  |
| White | 23026 (66.9%) | | 9429 (68.7%) | 32455 (67.4%) |  |
| Black | 4218 (12.3%) | | 1500 (10.9%) | 5718 (11.9%) |  |
| Parda | 6480 (18.8%) | | 2552 (18.6%) | 9032 (18.8%) |  |
| Missing | 0 | | 0 | 0 |  |
| **Sex** |  | |  |  | < 0.001^2^ |
| Female | 14204 (41.3%) | | 5265 (38.3%) | 19469 (40.4%) |  |
| Male | 20217 (58.7%) | | 8467 (61.7%) | 28684 (59.6%) |  |
| Missing | 0 | | 0 | 0 |  |
| **Underlying Disease** |  | |  |  | < 0.001^2^ |
| Diabetes | 7766 (22.6%) | | 2221 (16.2%) | 9987 (20.7%) |  |
| Hypertesion | 9714 (28.2%) | | 3451 (25.1%) | 13165 (27.3%) |  |
| Glomerulonephritis | 4456 (12.9%) | | 2778 (20.2%) | 7234 (15.0%) |  |
| Pyelonephritis | 790 (2.3%) | | 351 (2.6%) | 1141 (2.4%) |  |
| Other | 11695 (34.0%) | | 4931 (35.9%) | 16626 (34.5%) |  |
| Missing | 0 | | 0 | 0 |  |
| **Diabetes** |  | |  |  | < 0.001^2^ |
| No | 7766 (22.6%) | | 2221 (16.2%) | 9987 (20.7%) |  |
| Yes | 26655 (77.4%) | | 11511 (83.8%) | 38166 (79.3%) |  |
| Missing | 0 | | 0 | 0 |  |
| **Blood Group** |  | |  |  | < 0.001^2^ |
| A | 11511 (33.4%) | | 5440 (39.6%) | 16951 (35.2%) |  |
| AB | 1132 (3.3%) | | 596 (4.3%) | 1728 (3.6%) |  |
| B | 4064 (11.8%) | | 1770 (12.9%) | 5834 (12.1%) |  |
| O | 17714 (51.5%) | | 5926 (43.2%) | 23640 (49.1%) |  |
| Missing | 0 | | 0 | 0 |  |
| **Number Transfusion** |  | |  |  | 0.327^1^ |
| Mean (SD) | 0.41 (0.64) | | 0.41 (0.63) | 0.41 (0.64) |  |
| Median (Q1, Q3) | 0.00 (0.00, 1.00) | | 0.00 (0.00, 1.00) | 0.00 (0.00, 1.00) |  |
| Min - Max | 0.00 - 3.00 | | 0.00 - 3.00 | 0.00 - 3.00 |  |
| Missing | 0 | | 0 | 0 |  |
| **Pregnancy** |  | |  |  | < 0.001^2^ |
| No | 25507 (74.1%) | | 10615 (77.3%) | 36122 (75.0%) |  |
| Yes | 8914 (25.9%) | | 3117 (22.7%) | 12031 (25.0%) |  |
| Missing | 0 | | 0 | 0 |  |
| **Number of pregnancies** |  | |  |  | < 0.001^1^ |
| Mean (SD) | 2.14 (2.50) | | 1.78 (2.14) | 2.04 (2.41) |  |
| Median (Q1, Q3) | 2.00 (0.00, 3.00) | | 1.00 (0.00, 3.00) | 2.00 (0.00, 3.00) |  |
| Min - Max | 0.00 - 63.00 | | 0.00 - 41.00 | 0.00 - 63.00 |  |
| Missing | 20222 | | 8467 | 28689 |  |
| **Prior Transplant** |  | |  |  | < 0.001^2^ |
| Não | 29713 (86.3%) | | 12270 (89.4%) | 41983 (87.2%) |  |
| Sim | 4708 (13.7%) | | 1462 (10.6%) | 6170 (12.8%) |  |
| Missing | 0 | | 0 | 0 |  |
| **Number of Prior Transplant** |  | |  |  | < 0.001^1^ |
| Mean (SD) | 0.16 (0.42) | | 0.12 (0.37) | 0.15 (0.41) |  |
| Median (Q1, Q3) | 0.00 (0.00, 0.00) | | 0.00 (0.00, 0.00) | 0.00 (0.00, 0.00) |  |
| Min - Max | 0.00 - 5.00 | | 0.00 - 5.00 | 0.00 - 5.00 |  |
| Missing | 0 | | 0 | 0 |  |
| **Subregional** |  | |  |  | < 0.001^2^ |
| FUNDERP | 4725 (13.7%) | | 3066 (22.3%) | 7791 (16.2%) |  |
| UNICAMP | 2150 (6.2%) | | 1254 (9.1%) | 3404 (7.1%) |  |
| UNIFESP | 16871 (49.0%) | | 6295 (45.8%) | 23166 (48.1%) |  |
| HCFMUSP | 10675 (31.0%) | | 3117 (22.7%) | 13792 (28.6%) |  |
| Missing | 0 | | 0 | 0 |  |
| **cPRA class I (%)** |  | |  |  | < 0.001^1^ |
| Mean (SD) | 16.86 (31.43) | | 8.24 (21.08) | 14.40 (29.12) |  |
| Median (Q1, Q3) | 0.00 (0.00, 16.00) | | 0.00 (0.00, 0.00) | 0.00 (0.00, 9.00) |  |
| Min - Max | 0.00 - 100.00 | | 0.00 - 100.00 | 0.00 - 100.00 |  |
| Missing | 0 | | 0 | 0 |  |
| **Frequency HLA DR** |  | |  |  | < 0.001^1^ |
| Mean (SD) | 3.03 (1.96) | | 3.50 (1.75) | 3.17 (1.92) |  |
| Median (Q1, Q3) | 3.78 (1.15, 4.83) | | 4.14 (1.61, 5.06) | 3.96 (1.33, 4.83) |  |
| Min - Max | 0.00 - 6.25 | | 0.00 - 6.25 | 0.00 - 6.25 |  |
| Missing | 0 | | 0 | 0 |  |
| **Frequency HLA B** |  | |  |  | < 0.001^1^ |
| Mean (SD) | 1.14 (1.08) | | 1.29 (1.12) | 1.18 (1.10) |  |
| Median (Q1, Q3) | 0.81 (0.35, 1.68) | | 0.95 (0.42, 1.89) | 0.84 (0.36, 1.71) |  |
| Min - Max | 0.00 - 4.41 | | 0.00 - 4.41 | 0.00 - 4.41 |  |
| Missing | 0 | | 0 | 0 |  |
| **Frequency HLA A** |  | |  |  | < 0.001^1^ |
| Mean (SD) | 2.60 (2.75) | | 2.93 (2.88) | 2.70 (2.79) |  |
| Median (Q1, Q3) | 1.69 (0.77, 3.36) | | 2.08 (0.84, 4.62) | 1.76 (0.78, 4.20) |  |
| Min - Max | 0.00 - 17.64 | | 0.00 - 17.64 | 0.00 - 17.64 |  |
| Missing | 0 | | 0 | 0 |  |
| **HLA DR** |  | |  |  | < 0.001^2^ |
| heterozygous | 30069 (87.4%) | | 13199 (96.1%) | 43268 (89.9%) |  |
| heterozygous | 4352 (12.6%) | | 533 (3.9%) | 4885 (10.1%) |  |
| Missing | 0 | | 0 | 0 |  |
| **HLA B** |  | |  |  | < 0.001^2^ |
| heterozygous | 31999 (93.0%) | | 13099 (95.4%) | 45098 (93.7%) |  |
| heterozygous | 2422 (7.0%) | | 633 (4.6%) | 3055 (6.3%) |  |
| Missing | 0 | | 0 | 0 |  |
| **HLA A** |  | |  |  | < 0.001^2^ |
| heterozygous | 30679 (89.1%) | | 12492 (91.0%) | 43171 (89.7%) |  |
| heterozygous | 3742 (10.9%) | | 1240 (9.0%) | 4982 (10.3%) |  |
| Missing | 0 | | 0 | 0 |  |

1. Kruskal-Wallis rank sum test
2. Pearson’s Chi-squared test

cPRA: calculated panel class I; Anti-HBc: Hepatitis B surface antibody; HLA: Human leukocyte antigen
