# Supplementary material for: A machine learning prediction model for waiting time to kidney transplant
Source: PLoS One. 2021 May 20;16(5):e0252069. doi: 10.1371/journal.pone.0252069 (PMC8136711; doi:10.1371/journal.pone.0252069)
Supplement: S2 Table — (DOCX) [file pone.0252069.s002.docx]

**S2 Table. Sensitivity analysis of multivariate Cox model for each subregion (FUNDERP + UNICAMP), USP, and UNIFESP.**

|  | **(FUNDERP + UNICAMP)** | | | **USP** | | | **UNIFESP** | | |
| --- | --- | --- | --- | --- | --- | --- | --- | --- | --- |
| *Predictors* | *Estimates* | *CI* | *p* | *Estimates* | *CI* | *p* | *Estimates* | *CI* | *p* |
| Age between 18 and 60 ys (reference) | | | | | | | | | |
| Age more than 60 ys | 0.77 | 0.69 – 0.85 | **<0.001** | 0.70 | 0.63 – 0.78 | **<0.001** | 0.61 | 0.56 – 0.66 | **<0.001** |
| Age less than 18 ys | 3.90 | 3.30 – 4.61 | **<0.001** | 7.41 | 6.42 – 8.56 | **<0.001** | 5.38 | 4.83 – 6.00 | **<0.001** |
| Sex Male | 1.06 | 0.98 – 1.14 | 0.146 | 0.91 | 0.83 – 1.00 | **0.041** |  |  |  |
| Time on Dialysis [log] | 1.08 | 1.04 – 1.12 | **<0.001** | 1.07 | 1.02 – 1.12 | **0.003** | 1.02 | 0.99 – 1.06 | 0.134 |
| Blood group A (reference) | | | | | | | | | |
| Blood group AB | 1.10 | 0.91 – 1.32 | 0.315 | 1.32 | 1.09 – 1.61 | **0.006** | 1.33 | 1.14 – 1.54 | **<0.001** |
| Blood group B | 0.94 | 0.84 – 1.06 | 0.319 | 0.86 | 0.75 – 0.98 | **0.026** | 1.03 | 0.94 – 1.13 | 0.573 |
| Blood group O | 0.72 | 0.67 – 0.78 | **<0.001** | 0.56 | 0.51 – 0.62 | **<0.001** | 0.71 | 0.67 – 0.76 | **<0.001** |
| cPRA zero (reference) |  |  |  |  |  |  |  |  |  |
| cPRA between 0 and 50% | 0.72 | 0.65 – 0.79 | **<0.001** | 0.83 | 0.73 – 0.95 | **0.006** | 0.75 | 0.69 – 0.82 | **<0.001** |
| cPRA between 50 and 80% | 0.57 | 0.48 – 0.68 | **<0.001** | 0.60 | 0.50 – 0.73 | **<0.001** | 0.58 | 0.50 – 0.67 | **<0.001** |
| cPRA more than 80% | 0.20 | 0.16 – 0.25 | **<0.001** | 0.27 | 0.22 – 0.34 | **<0.001** | 0.21 | 0.17 – 0.25 | **<0.001** |
| Anti-HBc | 1.95 | 1.53 – 2.48 | **<0.001** | 2.25 | 1.75 – 2.89 | **<0.001** | 2.02 | 1.66 – 2.47 | **<0.001** |
| Frequency HLA DR | 1.10 | 1.08 – 1.13 | **<0.001** | 1.08 | 1.05 – 1.10 | **<0.001** | 1.06 | 1.04 – 1.07 | **<0.001** |
| Frequency HLA B | 1.11 | 1.07 – 1.14 | **<0.001** | 1.11 | 1.07 – 1.15 | **<0.001** | 1.12 | 1.09 – 1.15 | **<0.001** |
| Frequency HLA A | 1.03 | 1.02 – 1.04 | **<0.001** | 1.03 | 1.02 – 1.05 | **<0.001** | 1.06 | 1.05 – 1.07 | **<0.001** |
| HLA DR homozygous | 0.57 | 0.47 – 0.68 | **<0.001** | 0.31 | 0.24 – 0.40 | **<0.001** | 0.26 | 0.22 – 0.31 | **<0.001** |
| HLA B homozygous | 0.65 | 0.54 – 0.77 | **<0.001** |  |  |  | 0.90 | 0.78 – 1.03 | 0.119 |
| HLA A homozygous | 0.87 | 0.76 – 1.00 | **0.046** |  |  |  |  |  |  |
| Diabetes |  |  |  | 0.80 | 0.71 – 0.90 | **<0.001** |  |  |  |
| Prior Transplant |  |  |  | 0.84 | 0.73 – 0.97 | **0.018** | 0.91 | 0.82 – 1.01 | 0.086 |
| Observations | 8262 | | | 9943 | | | 16930 | | |
| R^2^ Nagelkerke | 0.289 | | | 0.432 | | | 0.397 | | |

cPRA: calculated panel class I; Anti-HBc: Hepatitis B surface antibody; HLA: Human leukocyte antigen
