# Supplementary material for: A machine learning prediction model for waiting time to kidney transplant
Source: PLoS One. 2021 May 20;16(5):e0252069. doi: 10.1371/journal.pone.0252069 (PMC8136711; doi:10.1371/journal.pone.0252069)
Supplement: S3 Table — (DOCX) [file pone.0252069.s003.docx]

**S3 Table.** **Competitive Risk Regression Model for Transplant considering the removal of the list or death as competitive events and the transplant as the outcome.**

|  | **Exp(coef)** | **Exp(-coef)** | **CI: 2.5%** | **CI: 97.5%** |
| --- | --- | --- | --- | --- |
| **HLA-DR frequency** | 1.081 | 0.925 | 1.047 | 1.115 |
| **HLA-B frequency** | 1.068 | 0.936 | 1.020 | 1.119 |
| **HLA-A frequency** | 1.032 | 0.969 | 1.014 | 1.052 |
| **Age more than 60 yrs** | 0.611 | 1.636 | 0.530 | 0.705 |
| **Age below 18 ys** | 4.596 | 0.218 | 3.679 | 5.742 |
| **Blood group AB** | 1.325 | 0.755 | 1.030 | 1.704 |
| **Blood group B** | 1.044 | 0.958 | 0.886 | 1.231 |
| **Blood group O** | 0.735 | 1.360 | 0.656 | 0.825 |
| **Previous Transplant** | 1.001 | 0.999 | 0.844 | 1.188 |
| **Subregion UNICAMP** | 0.780 | 1.281 | 0.629 | 0.969 |
| **Subregion UNIFESP** | 0.655 | 1.527 | 0.571 | 0.750 |
| **Subregion USP** | 0.537 | 1.862 | 0.461 | 0.625 |
| **cPRA between 0 and 50%** | 0.699 | 1.496 | 0.571 | 0.783 |
| **cPRA between 50 and 80%** | 0.590 | 1.695 | 0.456 | 0.763 |
| **cPRA more than 80%** | 0.367 | 2.723 | 0.280 | 0.482 |
| **Anti-HBc** | 3.179 | 0.345 | 2.313 | 4.368 |
| **Diabetes** | 1.222 | 0.818 | 1.056 | 1.414 |

Exp(coef): regression coefficient (OR); 2.5% and 97.5%: 95% confidence interval

cPRA: calculated panel class I; Anti-HBc: Hepatitis B surface antibody; HLA: Human leukocyte antigen
