# Supplementary material for: A machine learning prediction model for waiting time to kidney transplant
Source: PLoS One. 2021 May 20;16(5):e0252069. doi: 10.1371/journal.pone.0252069 (PMC8136711; doi:10.1371/journal.pone.0252069)
Supplement: S4 Table — (DOCX) [file pone.0252069.s004.docx]

**S4 Table.** **Simulated** **estimation of the probability of transplantation**

Table 1 shows the estimation of the probability of transplant using different pre-transplant predictors in 7 simulated cases. Table 2 shows the prediction of the probability of transplant at 12, 24, 36 and 48 months using the waiting list model.

**Table 01. Cox’s likelihood of transplant in simulated cases.**

| **Id** | **Age** | **Time On Dialysis** | **Blood Type** | **Prior Tx** | **Subregion** | **cPRA** | **Anti-HBc** | **Diabetes** | **DR.f** | **B.f** | **A.f** |
| --- | --- | --- | --- | --- | --- | --- | --- | --- | --- | --- | --- |
| 1 | 18 - 60 | 12 | O | No | FUNDERP | Zero | No | No | 3.9 | 0.84 | 1.76 |
| 2 | 18 - 60 | 12 | O | No | UNIFESP | Zero | No | No | 3.9 | 0.84 | 1.76 |
| 3 | 18 - 60 | 12 | O | No | HCFMUSP | Zero | No | No | 3.9 | 0.84 | 1.76 |
| 4 | 18 - 60 | 12 | O | No | FUNDERP | 0 - 50 | No | No | 3.9 | 0.84 | 1.76 |
| 5 | > 60 | 12 | O | No | UNIFESP | 50 - 80 | No | No | 3.9 | 0.84 | 1.76 |
| 6 | < 18 | 12 | O | No | UNIFESP | Zero | No | No | 3.9 | 0.84 | 1.76 |
| 7 | 18 - 60 | 12 | AB | No | UNIFESP | Zero | No | No | 3.9 | 0.84 | 1.76 |

Dr.f: HLA-DR frequency; B.f: HLA-B frequency; A.f: HLA-A frequency

cPRA: calculated panel class I; Anti-HBc: Hepatitis B surface antibody; HLA: Human leukocyte antigen

**Table 02. Probability of transplantation at a specific time point in simulated cases.**

| **Id** | **12 months** | **24 months** | **36 moths** | **48 moths** |
| --- | --- | --- | --- | --- |
| 1 | 13% | 25% | 36% | 45% |
| 2 | 9% | 17% | 25% | 32% |
| 3 | 7% | 13% | 19% | 25% |
| 4 | 10% | 20% | 28% | 36% |
| 5 | 4% | 8% | 11% | 15% |
| 6 | 39% | 64% | 79% | 87% |
| 7 | 15% | 28% | 40% | 49% |
