# Supplementary material for: A machine learning prediction model for waiting time to kidney transplant
Source: PLoS One. 2021 May 20;16(5):e0252069. doi: 10.1371/journal.pone.0252069 (PMC8136711; doi:10.1371/journal.pone.0252069)
Supplement: Attachment — Submitted filename: response_revisors.docx [file pone.0252069.s005.docx]

**Reviewer # 1:**

The manuscript presents interesting data on use of machine learning to predict time in waiting list. The authors have performed a model using several demographic data and test in a cohort of patients with success. They created a online live system to be tested.

**Thank you very much for your comments**

My minor concerns are:

- The intervals of age and cPRA seem pretty broad. Do they tested for more categories in-betweens?

**We fitted the cox model using cPRA and age at different cut-offs, which resulted in values of similar accuracy (c-index). Then we chose simple three-class values for age (< 18, between 18 and 60 and above 60 ys) and 4 class values for cPRA (zero, between o and 50%, between 50 and 80%, and above 80%). For age, these categories were chosen using the WHO cut-offs (children/older).**

- What about the influence of comorbities in this model: cardiovascular, bone, so on?

**We fitted the model with all variables available on the waiting list registration records. These variables are age, race, underlying diseases, cPRA, HLA, serology, and time on dialysis. Unfortunately, we did not have any comorbidities. The only comorbidity predictor available was diabetes. Diabetes was not an independent predictor of waiting time in the final model. A complete list of predictors is avaiable in the attachment table 01.**

- Can this model be tested outside Sao Paulo State and has the same fitness?

Probably so, provided some small adjustments are made in the model

**Reviewer # 2:**

The manuscript by de Andrade et al. is a well designed computational science project aimed at learning to predict the waiting time for deceased donor kidney transplant waiting list patients. Apparently, the output of their statistical models was the probability (hazard ratio) of the transplant, rather than the actual waiting time. Statistical methods predicting actual survival time exist or are possible in theory, and it would be nice to mention in introduction or discussion.

**Thank you very much for your comments**

**Yes, our model provides estimates on waiting time for a deceased kidney transplant using hazard ratio. However, it can also calculate the probability of a kidney transplant at a specific time point based on Cox survival time. We provided an attachment which shows the prediction of the probability of transplant at different time points in simulated cases (attachment 04).**

**We used the equations to calculate probabilities:**

**Hazard Ratio: The hazard ratio is the ratio of these two expected hazards: h0(t)exp (b1a)/ h0(t)exp (b1b) = exp(b1(a-b)) which does not depend on time, t. Thus the hazard is proportional over time**

**Probability of transplant at any time: proportion of units that survive beyond a specified time**

**Emil Hvitfeldt provided an excellent example using the Cox model to obtain the prediction of time and survival (https://github.com/tidymodels/censored)**

There are a few smaller details that can be polished in the manuscript. When saying that patients with missing data were excluded, please indicate: missing data in which (or maybe all?) variables.

**Data were excluded when subregional information was missing (n=51).** **We provided this information in the text.**

Also, please mention how did you use allelefrequencies.net database for calculating HLA antigen frequency. In particular, when alleles from the database were mapped to antigens, was care taken to account for broads and splits? Usually people refer to HLA dictionaries (such as the one here: https://pubmed.ncbi.nlm.nih.gov/19140825/) to make sure conversion from allelic to antigenic HLA types was correct.

**We clarifed HLA frequency calculation methods in the manuscript.**

**HLA frequency in the population of São Paulo state was obtained from the Allele Frequencies net database. To standardize HLA antigen assignments an HLA Dictionary [01] was used, and the serological equivalents were listed as expert assigned types.**

01. **Holdsworth R, Hurley CK, Marsh SG, Lau M, Noreen HJ, Kempenich JH, et al. The HLA dictionary 2008: a summary of HLA-A, -B, -C, -DRB1/3/4/5, and -DQB1 alleles and their association with serologically defined HLA-A, -B, -C, -DR, and -DQ antigens. Tissue Antigens. 2009;73(2):95-170. doi: 10.1111/j.1399-0039.2008.01183.x. PMID: 19140825**
